# Prevalence and spatial distribution patterns of human echinococcosis at the township level in Sichuan Province, China

**DOI:** 10.1186/s40249-021-00862-z

**Published:** 2021-06-05

**Authors:** Wei He, Li-Ying Wang, Wen-Jie Yu, Guang-Jia Zhang, Bo Zhong, Sha Liao, Qi Wang, Rui-Rui Li, Liu Yang, Ren-Xin Yao, Yang Liu, Zeli Danba, Sheng-Chao Qin, Shi-An Wang, Yan-Xia Wang, Yan Huang, Qian Wang

**Affiliations:** 1 Department of Parasitic Diseases, Sichuan Provincial Center for Disease Control and Prevention, No.6 Zhongxue Road, Chengdu, 610041 People’s Republic of China; 2 National Institute of Parasitic Diseases, Chinese Centre for Disease Control and Prevention, Chinese Centre for Tropical Diseases Research, WHO Collaborating Centre for Tropical Diseases, National Centre for International Research On Tropical Diseases, Ministry of Science and Technology, NHC Key Laboratory of Parasite and Vector Biology (National Institute of Parasitic Diseases, Chinese Center for Disease Control and Prevention), Shanghai, 200025 People’s Republic of China; 3 Doctorate School of Chemical and Biological Sciences for Health (CBS2), University of Montpellier, 34395 Montpellier, France; 4 Ganzi Prefectural Center for Disease Control and Prevention, No.139 Lucheng South Road, Ganzi Prefecture, 626000 People’s Republic of China; 5 Aba Prefectural Center for Disease Control and Prevention, No.178 Meigu Street, Aba Prefecture, 624000 People’s Republic of China; 6 Liangshan Prefectural Center for Disease Control and Prevention, Section 2 of Hangtian Avenue, Liangshan Prefecture, 615000 People’s Republic of China; 7 Ya’an Prefectural Center for Disease Control and Prevention, No.9 Fangcao Road, Daxing New District, Ya’an City, 625000 People’s Republic of China

**Keywords:** Echinococcosis, Prevalence, Spatial autocorrelation, Sichuan, China

## Abstract

**Background:**

Echinococcosis is a global zoonotic parasitic disease caused by *Echinococcus* larvae. This disease is highly endemic in Sichuan Province, China. This study investigates the prevalence and spatial distribution characteristics of human echinococcosis at the township level in Sichuan Province, geared towards providing a future reference for the development of precise prevention and control strategies.

**Methods:**

Human prevalence of echinococcosis was evaluated using the B-ultrasonography diagnostic method in Sichuan Province between 2016 and 2019. All data were collected, collated, and analyzed. A spatial distribution map was drawn to intuitively analyze the spatial distribution features. Eventually, the spatial autocorrelation was specified and local indicators of spatial association (LISA) clustering map was drawn to investigate the spatial aggregation of echinococcosis at the township level in Sichuan Province.

**Results:**

The prevalence of echinococcosis in humans of Sichuan Province was 0.462%, among which the occurrence of cystic echinococcosis (CE) was 0.221%, while that of alveolar echinococcosis (AE) was 0.244%. Based on the results of the spatial distribution map, a predominance of echinococcosis in humans decreased gradually from west to east and from north to south. The Global Moran’s *I* index was 0.77 (Z = 32.07, *P* < 0.05), indicating that the prevalence of echinococcosis in humans was spatially clustered, exhibiting a significant spatial positive correlation. Further, the findings of local spatial autocorrelation analysis revealed that the “high–high” concentration areas were primarily located in some townships in the northwest of Sichuan Province. However, the “low–low” concentration areas were predominantly located in some townships in the southeast of Sichuan Province.

**Conclusions:**

Our findings demonstrated that the prevalence of echinococcosis in humans of Sichuan Province follows a downward trend, suggesting that the current prevention and control work has achieved substantial outcomes. Nevertheless, the prevalence in humans at the township level is widely distributed and differs significantly, with a clear clustering in space. Therefore, precise prevention and control strategies should be formulated for clusters, specifically strengthening the “high–high” clusters at the township level.

**Graphic Abstract:**

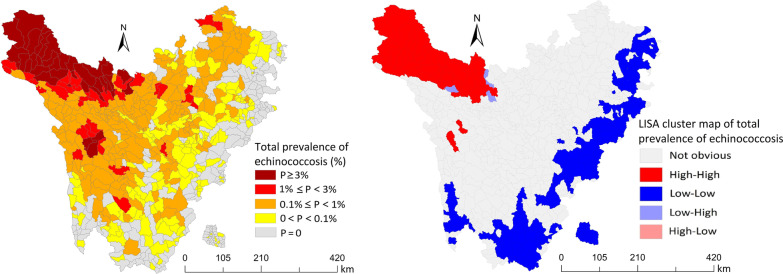

## Background

Echinococcosis (also known as hydatid disease) is a zoonotic parasitic disease caused by the larvae of *Echinococcus* spp. It belongs to group C infectious diseases in China due to its specific manifestation and transmission mode in hosts. Due to its severity, echinococcosis is a global public health problem [1–3]. Previous studies reported that four species of the genus *Echinococcus*, including *E. granulosus*, *E. multilocularis*, *E. oligarthrus*, and *E. vogeli*, are responsible for echinococcosis in humans; and *E. granulosus* and *E. multilocularis* are the most significant [4, 5]. Larvae of these four species are subjected to three types of echinococcosis in humans, including cystic echinococcosis (CE), alveolar echinococcosis (AE), and polycystic echinococcosis (PE) [6]. Infected canines and felines including dogs, wolves, foxes, and cats are important infection sources of echinococcosis. Intermediate hosts include even-toed ungulates and small mammals, while humans are implicated as incidental hosts. This disease is primarily transmitted via the fecal–oral route. Individuals living in echinococcosis-endemic areas are susceptible to echinococcosis, and high-risk groups primarily include those involved in agricultural production, livestock slaughter, fur processing, and hunting. The life cycle of *Echinococcus* spp. must be completed by canine (feline) and cloven-hoofed animal hosts/small mammals, where three processes including egg, metacestode, and adult worm are involved. This disease has clinically characterized symptoms, including cyst pressure and irritation to affected organs, pain, fever, and allergic reactions [7]. Studies indicate that the mortality rate of AE exceeds 90% in patients either untreated or undertreated for 10–15 years [8].

Echinococcosis is globally widespread, showing priority in agricultural and pastoral areas. Different *Echinococcus* spp. causing different types of echinococcosis exhibits different global distributions, causing differences in the prevalence of different types of echinococcosis and regions. Only two types of echinococcosis (CE and AE) have been reported in China. The global disease burden of AE in terms of disability-adjusted of life years (DALY) in China accounts for 91%, while that of CE accounts for 40% [9, 10]. Multiple recent reports indicate that echinococcosis is primarily dominant in pastoral, semi-agricultural, and semi-pastoral areas of Inner Mongolia, Sichuan, Tibet, Gansu, Qinghai, Ningxia, Yunnan, Shaanxi, and Xinjiang provinces/autonomous regions in northwest China, specifically in Sichuan, Tibet, and Qinghai in Qinghai-Tibet Plateau [11, 12]. Of these regions, Sichuan is one of the provinces with the most severe prevalence of echinococcosis in China, with a mixed epidemic area of CE and AE. Based on a report by the national survey conducted in 2012, echinococcosis was prevalent in 35 counties in Sichuan, mainly predominant in the whole area of Ganzi (18 counties) and Aba prefectures (13 counties), in Muli and Yuexi counties of Liangshan prefecture, also in Tianquan and Baoxing counties of Ya'an city. Subsequently, the estimated prevalence was 1.08% [13, 14].

Although the occurrence of echinococcosis in Sichuan Province is clear at the county level [14], it remains elusive at the township level and warrants further inquiry. Herein, we performed echinococcosis screening for the whole population in Sichuan Province between 2016 and 2019 to unravel the current status of the human echinococcosis prevalence.

The spatial statistical analysis method has been widely applied in the field of epidemiologic study of echinococcosis, specifically in evaluating the difference of prevalence in different regions and identifying disease clustering [15, 16]. For instance, Brundu et al. analyzed the spatial scan statistics of 1029 pastures where CE of bovine was discovered in Sardinia and Italy, revealing two clusters [17]. Elsewhere, a spatial autocorrelation analysis of echinococcosis prevalence in 13 counties of Aba Prefecture in Sichuan Province was conducted using the global Moran’s *I* method by Qi demonstrating significant clustering distribution in echinococcosis prevalence. However, the global Getis'G result elucidated clusters with a high prevalence of echinococcosis [18]. Additionally, Zhao et al. used spatial scan statistics and spatial autocorrelation analysis methods to assess the detection rate of echinococcosis in 18 counties of Ganzi prefecture in Sichuan Province, and reported aggregation in the spatial distribution of echinococcosis [19].

This study analyzed the spatial aggregation of human echinococcosis prevalence at the township level in Sichuan Province using global and local spatial autocorrelation analysis methods. This was geared towards identifying spatial aggregation areas of echinococcosis at the township level in Sichuan Province, to establish the areas that should strengthen the key prevention and control in the future. Besides, we purposed to provide a baseline reference for the formulation of precise strategies and measures for the prevention and mitigation of echinococcosis.

## Methods

### Survey areas

**Fig. 1:**
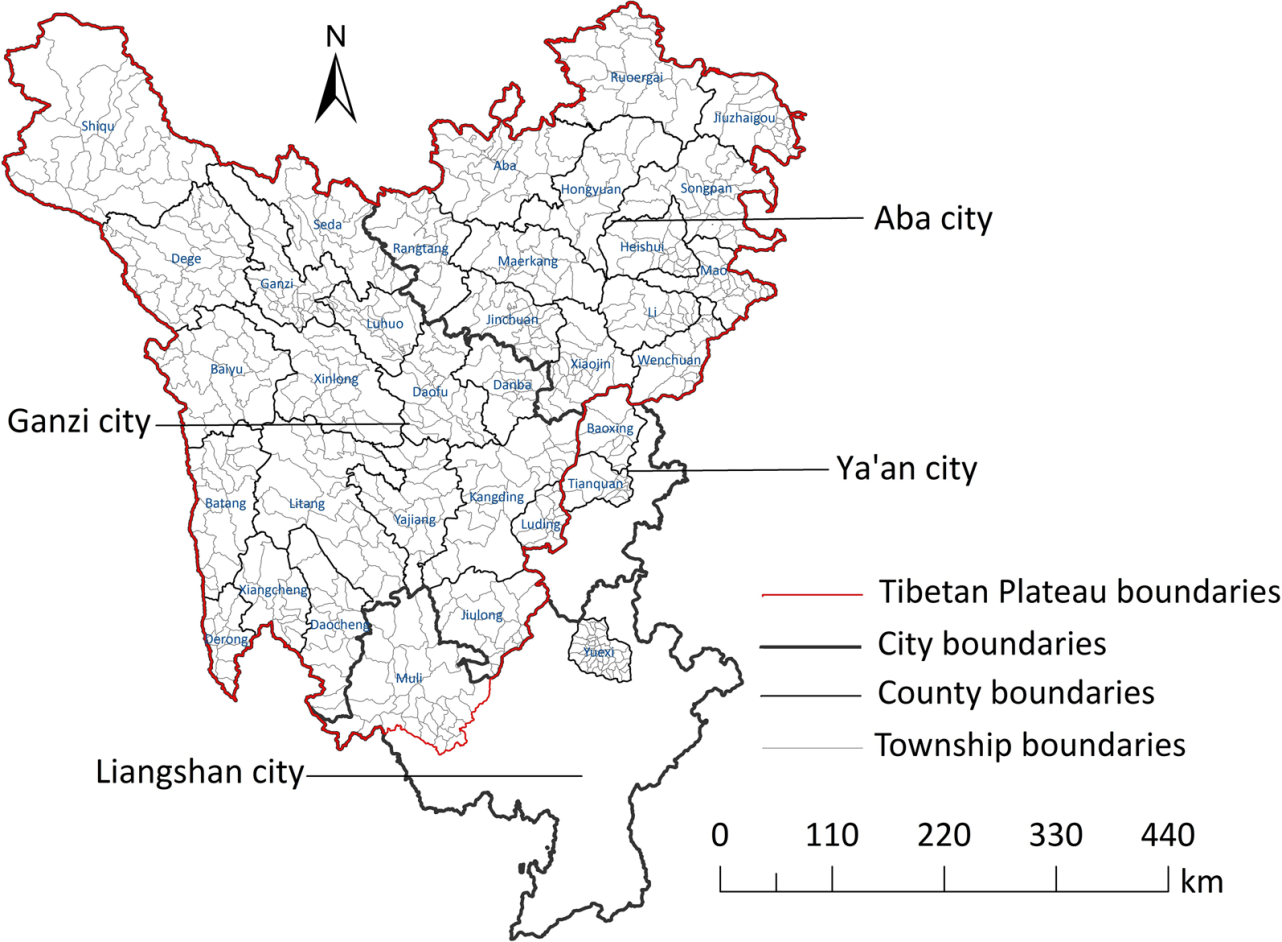
Map of townships for echinococcosis screening in Sichuan Province

A survey conducted between 2016 and 2019 was used to investigate the prevalence of human echinococcosis in areas where many echinococcosis cases were previously reported. The scope of this study was full coverage comprising 325 townships under 18 counties in Ganzi prefecture, 230 townships under 13 counties in Aba prefecture, 70 townships under 2 counties in Liangshan prefecture, and 24 townships under two counties in Ya’an city. Overall, 649 townships were covered to screen human echinococcosis (Fig. 1). Among the 35 endemic counties of echinococcosis in Sichuan Province, except Tianquan, Baoxing, and Yuexi counties, the rest counties belong to the Qinghai-Tibet Plateau.

### Survey contents and methods

#### Target population

All permanent residents (including those who had a continuous residence at the survey areas for more than 6 months) aged 2 years and above were screened for echinococcosis.

#### Survey of prevalence

The prevalence of human echinococcosis was investigated based on the requirements of the Technical Plan for Echinococcosis Prevention and Control [20]. A B-ultrasound examination of the abdomen was performed using a portable ultrasonic diagnostic instrument. Patients were diagnosed following the Diagnostic Criteria for Echinococcosis (WS257-2006) [21], while serological tests were supplemented to the suspected patients. Specifically, the anti-echinococcosis antibody in the serum of suspected patients was detected using ELISA. The IgG Antibody Diagnostic Kit was purchased from Shenzhen Kangbaide Biotechnology Co., Ltd., China.

#### Spatial clustering analysis

The detection rate of human echinococcosis in Sichuan Province was analyzed by global autocorrelation and local control autocorrelation to understand the aggregation degree and scope of echinococcosis. Based on the literature, spatial autocorrelation accurately reflected the aggregation degree of an indicator in a spatial unit. Spatial autocorrelation analysis included global spatial autocorrelation and local spatial autocorrelation analyses. Spatial autocorrelation coefficients are often used to quantitatively judge the spatial autocorrelation form and correlation size. Several spatial autocorrelation coefficients suitable for different data types have been reported. For instance, the statistics of common adjacent edges are suitable for type variables, while the statistics of Moran’s *I*, Geary’s *C,* and Getis *G* are primarily suitable for numerical variables. Since the prevalence of echinococcosis was a numerical variable, Moran’s *I* statistics were used for analysis [22].

#### Global spatial autocorrelation analysis

Global spatial autocorrelation analysis was used to analyze whether the specified attributes in the whole research scope exhibit autocorrelation and global Moran’s *I* index can be used for global spatial autocorrelation analysis, and the formula is as follows:

$$I = \frac{{n\sum\limits_{i = 1}^{n} {\sum\limits_{j = 1}^{n} {w_{ij} \left( {x_{i} - \overline{x}} \right)\left( {x_{j} - \overline{x}} \right)} } }}{{\sum\limits_{i = 1}^{n} {\sum\limits_{j = 1}^{n} {w_{ij} } \sum\limits_{i = 1}^{n} {\left( {x_{i} - \vec{x}} \right)^{2} } } }}$$

where *n* refers to the total number of observed values, and ${x}_{i}$ refers to the observed value at position *i*. ${x}_{j}$ is the observed value at position *j*, $i\ne j$. $\overline{x }$ refers to the average of observed values at all *n* positions, $\overline{x }=\frac{1}{n}\sum_{i=1}^{n}{x}_{i}$*.* ${w}_{ij}$ refers to the element value of symmetric binomial distribution spatial weight matrix, a measure of the influence and action between spatial positions *i* and *j*.

Moran’s *I* range from − 1 to $+$ 1. Moran’s *I* index greater than 0 indicates that ${x}_{i}$ and ${x}_{j}$ change in the same direction, and the data show positive autocorrelation. The closer the value was to $+$ 1, it indicated that similar observed values gather in a similar region, and the stronger the positive correlation, the higher the regional aggregation. Moran’s *I* index was less than 0, suggesting that ${x}_{i}$ and ${x}_{j}$ change in different directions and the data were negatively correlated. The closer the value was to − 1, the stronger the negative correlation when different observed values were gathered; Moran’s *I* index was close to 0, indicating that the observed values were mostly randomly distributed in space with no spatial autocorrelation.

Global spatial autocorrelation analysis was used to establish whether the prevalence of echinococcosis exists aggregation in the whole space of Sichuan Province. Only with aggregation in the whole space, local spatial autocorrelation analysis was carried out.

#### Local spatial autocorrelation analysis

Local spatial autocorrelation analysis was used to analyze whether the attributes specified in specific local locations harbored autocorrelation. Therefore, based on global spatial autocorrelation analysis, local spatial autocorrelation analysis was used to establish the spatial autocorrelation of each township, and make clear the specific spatial location and significance of aggregation. The local indicators of spatial association (LISA) included Moran’s *I* and Getis *G* statistics, and the most commonly used local Moran’s *I* statistics were applied. Local Moran’s *I* statistic was used to reveal whether the observed variables were clustered in local areas, and the formula is:

$$I_{i} = \frac{{y_{i} - \overline{y}}}{{\delta^{2} }}\mathop \sum \limits_{j = 1}^{n} W_{ij} \left( {y_{j} - \overline{y}} \right)$$

In the formula, ${\delta }^{2}=\frac{1}{n}\sum_{i=1}^{n}{\left({y}_{i}-\overline{y }\right)}^{2}$, $\overline{y }=\frac{1}{n}\sum_{i=1}^{n}{y}_{i}$

Local Moran’s* I* > 0 indicates that spatial units with similarly observed variables are clustered in space, suggesting that a high-value unit was surrounded by units with similar high value (high–high), or a low-value unit was surrounded by units with similar low value (low–low). This phenomenon is also called “clustering”. Moran’s* I* < 0 indicates that units with dissimilar observed values were clustered in space, and a low value surrounded by a high value (low–high), or a high value surrounded by a low value (high–low). This phenomenon is also called “outlier”.

LISA clustering map directly displays aggregation areas and aggregation types. Different colors of small areas represent different local correlation types. Red indicated “high–high” aggregation areas, dark blue indicated “low–low” areas, magenta indicated “high–low” areas, and light blue depicted “low–high” areas [23].

### Data processing and statistical analysis

All data (comprising name, sex, age, lesion type, lesion size, and location) were inputted using the software Epi Info 7.2.4 (Department of Health & Human Services, USA). Data of 35 counties were subsequently combined. The error and duplicate data were eliminated, whereby the error was corrected via the double-entry comparison method, and error corrections were made using the two-item comparison method, and eventually a human echinococcosis screening database was created.

Statistical data analyses were executed using the statistical software R 3.0.3 (Lucent Technologies, Jasmine Mountain, USA). We also calculated the prevalence of human echinococcosis in each township.

The prevalence of human echinococcosis was calculated as per the following formula:

$$P = n/N \times 100\%$$

where *P* is the prevalence of the population in the surveyed area, *n* refers to the number of patients detected, and *N* denotes the number of examined people.

ArcGIS 10.3 software for Desktop (Environmental Systems Research Institute, USA) was used to map the spatial distribution map of the human echinococcosis prevalence at the township level and analyze the trend surface, while the GeoDa 1.6.7 software (Center for Spatial Data Science, University of Chicago) was adopted to analyze the prevalence of human echinococcosis in townships for global and local spatial autocorrelations. The spatial autocorrelation indexes were respectively adopted as Global Moran’s *I* index and Anselin’s Local Moran’s *I* index, and the related results were visually displayed. A level of *P* < 0.05 was considered statistically significant.

## Results

### Basic information

In total, 649 townships from 35 endemic counties in 4 prefectures/cities of Sichuan Province were recruited to carry out this survey. A total of 2 758 525 people were examined, and 11 743 patients were detected. Notably, the distribution of patients was found in 426 townships, including 405 townships with CE patients, 167 townships with AE patients, and 146 townships with both kinds of echinococcosis patients (Table 1).

**Table 1:** Distribution of human echinococcosis at the township level in Sichuan Province

City/prefecture	Number of townships surveyed	Number of CE epidemic townships	Number of AE epidemic townships	Number of total epidemic townships
Aba prefecture	230	134	40	146
Ganzi prefecture	325	255	125	262
Liangshan Prefecture	70	12	0	12
Ya’an city	24	4	2	6
Total	649	405	167	426

*AE* alveolar echinococcosis, *CE* cystic echinococcosis

**Fig. 2:**
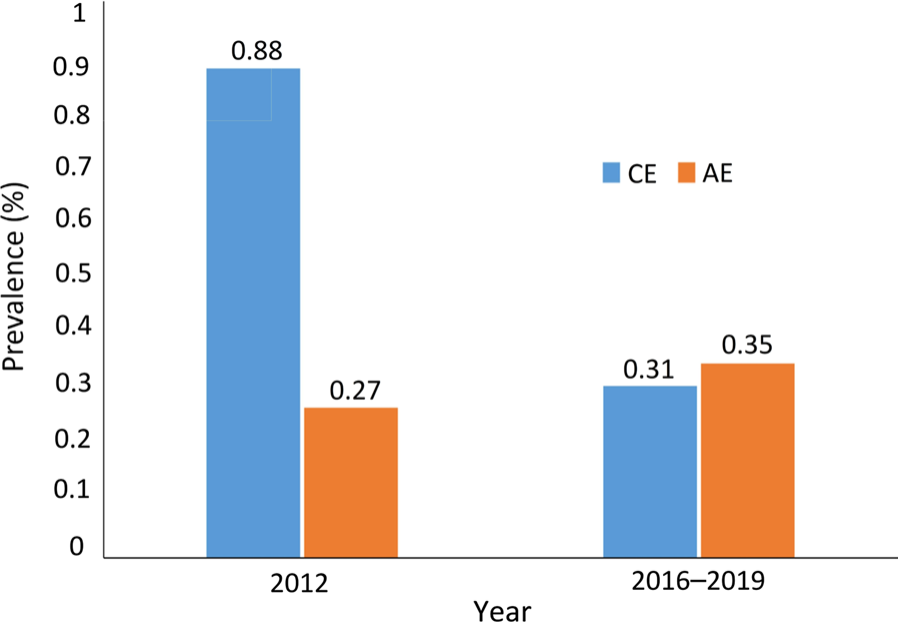
The comparison of the prevalence of cystic echinococcosis and alveolar echinococcosis in 2012 and 2016–2019. *AE* alveolar echinococcosis, *CE* cystic echinococcosis

Overall, the survey conducted in 2016–2019 demonstrated that the prevalence of AE (0.351%) was significantly higher than that of CE (0.310%) (*χ*^2^ = 28.166, *P* < 0.05). However, the survey findings of 2012 revealed that the prevalence of CE (0.881%) was significantly higher than that of AE (0.273%) [14] (*χ*^2^ = 372.204, *P* < 0.05, Fig. 2).

### Geographical distribution

#### Spatial distribution characteristics

The prevalence of echinococcosis in Sichuan Province was 0.462% (see Table 2). Noticeably, 5 out of the 35 endemic counties exhibited a predominance of more than 1%, including Shiqu (6.512%), Seda (3.056%), Baiyu (1.237%), Dege (1.180%), and Ganzi (1.171%) counties.

**Table 2:** Prevalence of human echinococcosis of epidemic counties in Sichuan Province

City/prefecture	County	Total number of townships	Population in endemic areas	Number of exam'd	Total number of patients	Total prevalence (%)	CE		AE	
							Number of patients	Prevalence (%)	Number of patients	Prevalence (%)
Aba Prefecture	Aba	21	84 299	75 150	109	0.145	83	0.110	26	0.035
	Heishui	17	63 700	57 226	46	0.080	39	0.068	7	0.012
	Hongyuan	11	47 186	45 751	90	0.197	83	0.181	7	0.015
	Jinchuan	23	86 657	79 499	95	0.119	91	0.114	4	0.005
	Jiuzhaigou	19	80 701	77 204	8	0.010	8	0.010	0	0.000
	Li	13	50 235	45 405	17	0.037	17	0.037	0	0.000
	Maerkang	14	55 459	50 378	160	0.318	160	0.318	0	0.000
	Mao	21	111 578	106 125	5	0.005	5	0.005	0	0.000
	Rangtang	12	44 525	39 564	207	0.523	3	0.008	204	0.516
	Ruoergai	19	80 931	71 028	380	0.535	380	0.535	0	0.000
	Songpan	26	70 087	65 888	2	0.003	2	0.003	0	0.000
	Wenchuan	13	100 093	96 302	2	0.002	2	0.002	0	0.000
	Xiaojin	21	82 243	76 227	30	0.039	25	0.033	5	0.007
Ganzi Prefecture	Batang	19	52 232	48 108	52	0.108	52	0.108	0	0.000
	Baiyu	17	59 118	55 382	685	1.237	423	0.764	264	0.477
	Danba	15	54 842	53 538	25	0.047	24	0.045	1	0.002
	Daofu	22	44 549	40 588	109	0.269	109	0.269	0	0.000
	Daocheng	14	35 393	28 206	16	0.057	15	0.053	1	0.004
	Derong	12	28 785	26 490	3	0.011	4	0.015	0	0.000
	Dege	26	90 523	81 810	965	1.180	698	0.853	269	0.329
	Ganzi	22	66 501	62 016	726	1.171	283	0.456	450	0.726
	Jiulong	18	53 471	52 770	13	0.025	13	0.025	0	0.000
	Kangding	21	117 695	112 059	68	0.061	42	0.037	27	0.024
	Litang	24	72 344	68 346	265	0.388	265	0.388	0	0.000
	Luhuo	16	39 890	39 768	108	0.272	103	0.259	5	0.013
	Luding	12	87 961	85 978	18	0.021	13	0.015	5	0.006
	Seda	17	56 777	51 403	1571	3.056	739	1.438	840	1.634
	Shiqu	22	89 367	88 116	5738	6.512	1731	1.964	4061	4.609
	Xiangcheng	12	29 839	26 936	9	0.033	9	0.033	0	0.000
	Xinlong	19	51 970	46 209	166	0.359	147	0.318	19	0.041
	Yajiang	17	42 034	38 577	30	0.078	30	0.078	0	0.000
Liangshan Prefecture	Muli	29	138 555	121 198	12	0.010	12	0.010	0	0.000
	Yuexi	41	378 425	349 851	7	0.002	7	0.002	0	0.000
Ya’an City	Baoxing	9	51 725	43 940	2	0.005	1	0.002	1	0.002
	Tianquan	15	158 835	135 099	4	0.003	3	0.002	1	0.001
Total		649	2 758 525	2 542 135	11 743	0.462	5621	0.221	6197	0.244

*AE* alveolar echinococcosis, *CE* cystic echinococcosis

Based on the analysis at the township level, the top three townships with the highest overall prevalence were all under the jurisdiction of Shiqu county, including Changsha Gongma (14.961%), Gemeng (12.065%), and Sexu (10.588%) townships. Specifically, the top three townships with the highest prevalence of CE were Gayi (3.243%) and Changxu Gongma (3.210%) townships in Shiqu county and Suoba (3.220%) township in Dege county. Moreover, the top three townships with the highest prevalence of AE were Changsha-Gongma (12.986%), Gemeng (10.462%), and Mengyi (10.363%) townships in Shiqu county. From Table 3, the number of townships with human prevalence 0–0.1%, 0.1–1% and 1% or above was 153, 209, and 64, respectively.

**Table 3:** Prevalence of different types of echinococcosis in Sichuan Province

Human prevalence (%)	Number of CE epidemic townships	Number of AE epidemic townships	Number of total epidemic townships
≥ 1	47	36	64
0.1–1	197	71	209
< 0.1	161	60	153
Total	405	167	426

*AE* alveolar echinococcosis, *CE* cystic echinococcosis

**Fig. 3:**
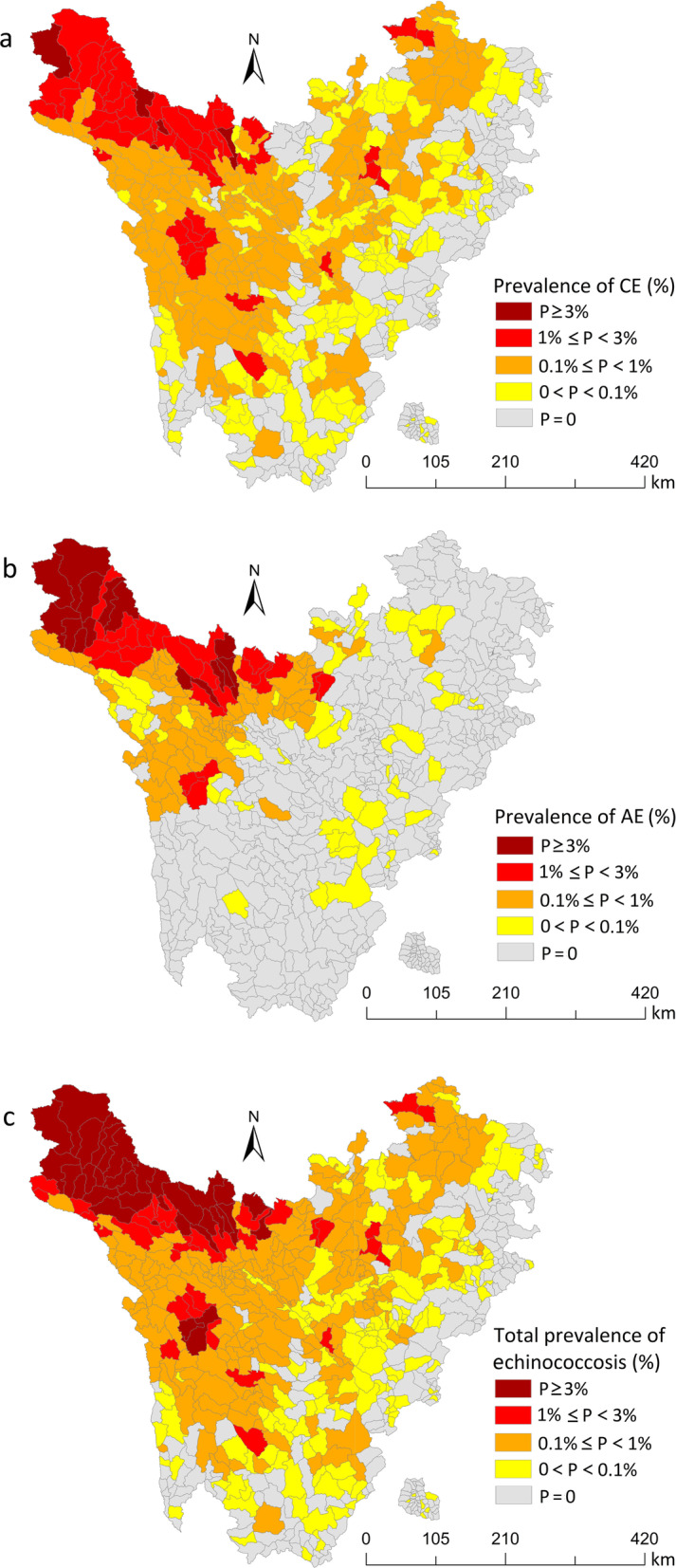
Distribution of the prevalence of echinococcosis in Sichuan Province. **a** Distribution of cystic echinococcosis; **b** Distribution of alveolar echinococcosis; **c** Distribution of total prevalence of echinococcosis. *AE* alveolar echinococcosis, *CE* cystic echinococcosis

The spatial distribution map of Sichuan Province displayed that the high prevalence of echinococcosis was primarily distributed in the west, northwest, and north, while the low prevalence was predominantly distributed in the south and east. The prevalence of echinococcosis in Sichuan Province exhibited spatial clustering characteristics, indicating that areas with high or low prevalence seemingly aggregated into pieces. Nevertheless, the judgment of this aggregation was statistically insignificant, hence spatial autocorrelation analysis and other methods were adopted for further inference (Fig. 3).

#### Spatial clustering analysis

Global spatial autocorrelation analysis results of echinococcosis prevalence at the township level in Sichuan Province indicated positive spatial autocorrelation and aggregation distribution rather than random distribution (Table 4).

**Table 4:** Spatial autocorrelation global Moran's *I* analysis on the prevalence of echinococcosis

Prevalence of echinococcosis	Moran's *I* index	Z-value	*P*-value
Total	0.77	22.07	< 0.01
CE	0.70	29.67	< 0.01
AE	0.71	30.52	< 0.01

*AE* alveolar echinococcosis, *CE* cystic echinococcosis

**Fig. 4:**
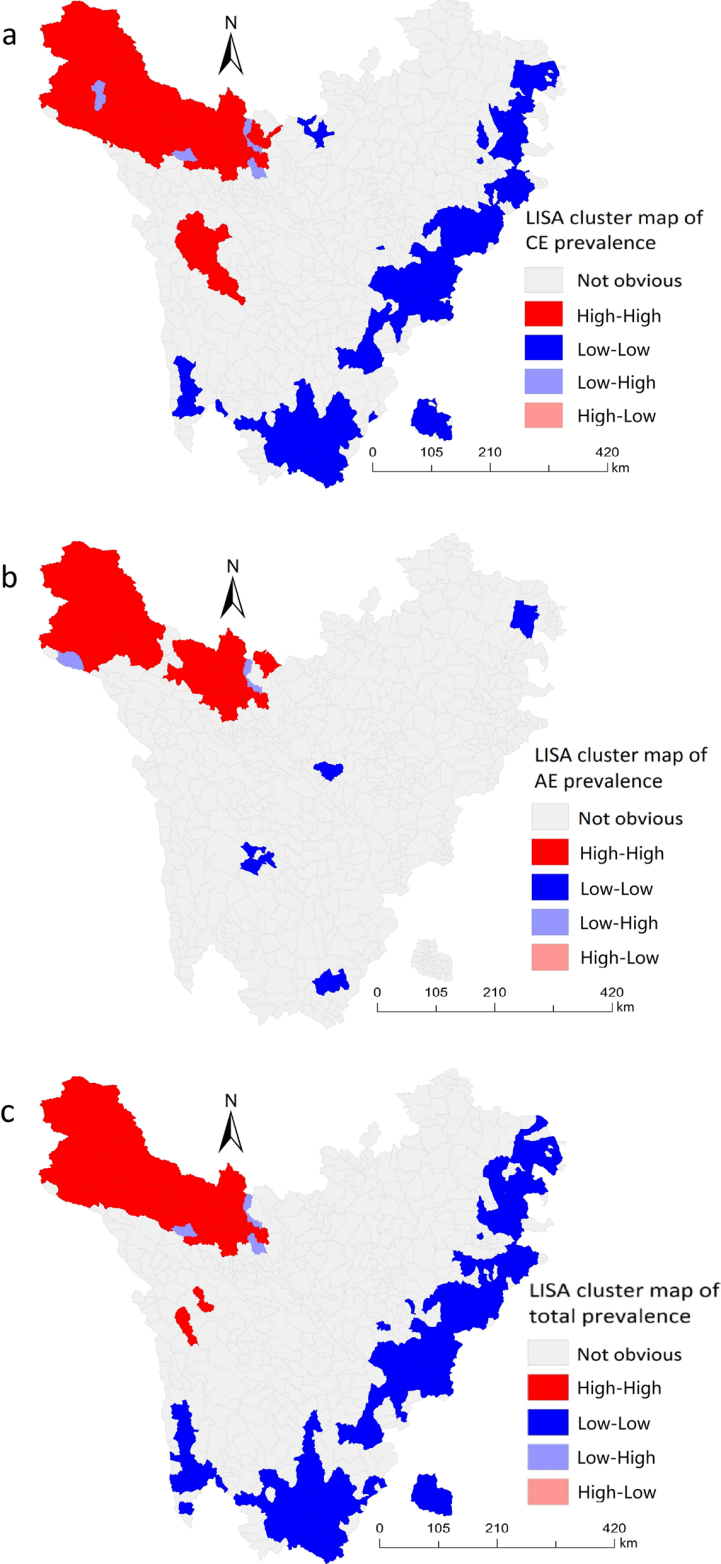
Local indicators of spatial association (LISA) clustering map of new prevalence of echinococcosis in the rural population of Sichuan Province. **a** LISA cluster map of CE. **b** LISA cluster map of AE. **c** LISA cluster map of total prevalence of echinococcosis

In this subsection, the outcomes of local spatial autocorrelation analysis revealed that the “high–high” gathering areas of overall prevalence were mainly located in the townships of Shiqu, Seda, Ganzi, Dege, and Baiyu counties, totaling 41. The “high–high” gathering areas of CE prevalence were dominantly located in townships of Shiqu, Seda, Ganzi, Dege, Baiyu, Rangtang, Xinlong, and Litang counties, totaling 48. The “high–high” gathering areas of AE prevalence were predominantly located in towns of Shiqu, Dege, Seda, Ganzi, and Rangtang counties, totaling 31. The “low–low” gathering areas of the overall prevalence were primarily located in townships of Jiuzhaigou, Songpan, Heishui, Mao, Li, Wenchuan, Xiaojin, Baoxing, Tianquan, Kangding, Luding, Muli, Daocheng, Xiangcheng, and Yuexi counties, totaling 193. The “low–low” gathering areas of the predominance of CE were largely located in townships of Jiuzhaigou, Songpan, Heishui, Mao, Li, Wenchuan, Xiaojin, Baoxing, Tianquan, Kangding, Luding, Muli, Daocheng, Xiangcheng, and Yuexi counties, totaling 165. Eventually, the “low–low” gathering areas of the occurrence of AE were generally in the townships of Jiuzhaigou, Songpan, and Kangding, totaling 4 (Fig. 4).

## Discussion

Echinococcosis is a global zoonotic parasitic disease that seriously threatens the health and life safety of people. It impacts social and economic development, causing heavy economic burdens to families. It is one of the major reasons for poverty among the population in endemic areas of China [24]. Here, a survey was performed in 35 epidemic counties previously identified in 2012 to understand human echinococcosis prevalence in Sichuan Province. We specifically investigated the epidemic areas and human echinococcosis at the township level in Sichuan Province. Our results reveal that the echinococcosis patients were distributed in 426 townships, primarily located in the northwestern part of Sichuan Province and concentrated in high mountain meadows, pastoral areas with a cold, arid climate, little rainfall, semi-agricultural, and semi-pastoral areas. This is attributed to *Echinococcus* eggs adapting to the natural environments of cold, dry, and rainless [25, 26]. Additionally, we noted abundant animal resources in these environments, forming a relatively suitable food chain for predation and prey, thus enabling *Echinococcus* spp. to form a complete life history, resulting in echinococcosis prevalence [27, 28]. CE and AE were prevalent in 405 and 167 townships; CE epidemic areas were more widespread than AE. This observation is potentially attributed to the fact that CE is primarily spread in the biological circulation chain comprising domestic dogs as the main definitive host and domestic animals as intermediate hosts [29, 30], However, AE is primarily spread in the biological circulation chain composed of foxes and dogs as the main definitive host and small mammals as intermediate hosts [31]. In contrast, the distribution range of small mammals is smaller than that of domestic animals [32].

Based on the results of the 2012 national echinococcosis prevalence survey, the number of patients with CE in Sichuan Province was distinctively higher than those with AE, the findings of the 2016–2019 survey revealed that AE patients accounted for 52.77% of the total number of patients. The major reasons for this discrepancy may be as follows. Firstly, the infectious source of CE was primarily dogs. Besides, since the implementation of the Echinococcosis Prevention and Control Project, Sichuan Province had standardized the management and deworming of dogs, and thus the number of stray dogs reduced; the infectious source was effectively controlled, thereby decreasing the prevalence of echinococcosis [33]. Secondly, the surgical operation difficulty of CE was less compared to that of AE, and more CE patients were effectively cured [34, 35]. Thirdly, the experience and diagnostic techniques of ultrasonographers differed from one region to another, potentially leading to inaccurate diagnosis.

The predominance of human echinococcosis in Sichuan Province followed a trend in both the east–west and north–south directions for most of the years, gradually decreasing from west to east and from north to south. These findings are consistent with those of trend surface analysis of echinococcosis conducted in Aba Prefecture in 2013 [18] and with the spatial and temporal distribution characteristics of the new human echinococcosis prevalence in Sichuan Province between 2007 and 2017 [36]. The explanations why areas with high prevalence were essentially distributed in the northwest of Sichuan Province may be as follows. Firstly, the northwestern part of Sichuan Province is characterized by the natural environment of high altitude, dry with little rainfall, low temperature, low humidity, low oxygen content, long sunshine time, and most shrub vegetation types are bushes, these factors favor the *Echinococcus* eggs survival for a longer time, thus exacerbating the spread of echinococcosis [37, 38]. Secondly, studies on the Qinghai-Tibet Plateau in northwest Sichuan Province revealed that ecological changes caused by overgrazing of grassland promoted the spread of multilocular echinococcosis. The overgrazing of grassland decreased the grass density and height, which is beneficial to the habitat formation of small mammals including *Microtus fuscus* and plateau pika, this significantly increases the number and density of these small mammals. Furthermore, a large number of owned dogs and stray dogs were noted in these areas, this is conducive to the transmission and circulation of *E. multilocularis*, causing a high prevalence of AE [39, 40]. Thirdly, a large number of domestic animals as intermediate hosts of CE increased the spread of echinococcosis, and the grassland was a favorable environment for the intermediate hosts (small mammals) of AE [39, 41, 42]. Fourthly, some analysis of risk factors for echinococcosis transmission carried out in Sichuan showed that the large number of dogs per household, the high infection rate of dogs, the high density of dog feces in the household courtyard, the high density of local small mammals, the low awareness rate of prevention and control knowledge in herdsmen, the higher incidence of echinococcosis in aged people, the traditional pastoral nomadic lifestyle, the large numbers of bovine and sheep/goat were all related to the population suffering from echinococcosis [43, 44]. previous findings showed that the northwestern part of Sichuan Province including Shiqu, Seda, Ganzi, and Baiyu counties had numerous livestock, that could be raised for a long time since local farmers were not willing to kill them. Most areas lacked centralized slaughterhouses or standardized slaughter management, which might be one of the reasons for the higher human prevalence rate in these areas than that in the southeast [45]. Fifthly, many types of wild animals with large quantities were reported in the northwest of Sichuan Province, therefore, snow disasters, lack of enough food in spring, and other reasons caused the death of livestock and wild animals. Consequently, this caused the formation of the circulation chain of echinococcosis transmission, thus aggravating the transmission of echinococcosis [46, 47]. Sixthly, illiteracy among most residents of Tibetan areas in the northwest of Sichuan Province, with the bad production and living habits including close contact with dogs, feeding sick cattle (sheep) viscera to dogs, not washing hands after eating, and lack of safe drinking water sources, significantly increased the risk of transmission infection [47, 48].

Numerous reports indicate that the prevalence and transmission of echinococcosis are influenced by natural, biological, and social factors; restricted by the adult worms in definitive host segments and eggs in the external environment; larvae in intermediate hosts and stability of parasites with spatial autocorrelation [35, 49–54].

We employed spatial autocorrelation statistical analysis to explore the spatial distribution characteristics of echinococcosis at the township level in Sichuan Province. Through global autocorrelation analysis of echinococcosis prevalence, and the prevalence of populations CE and AE, the predominance of different types of echinococcosis exhibited a positive spatial correlation, with an aggregated distribution.

Moreover, global spatial autocorrelation analysis results showed that echinococcosis prevalence was clustered in space with a positive spatial correlation. On the other hand, in the local spatial autocorrelation analysis, the LISA aggregation map showed “high–high” and “low–low” clusters of human echinococcosis prevalence at the township level. Notably, the “high–high” clusters were dominantly distributed in most of the townships of Shiqu, Seda, Ganzi, Dege, and Baiyu counties near the northwest of Sichuan Province, this is consistent with the spatial aggregation analysis of echinococcosis conducted in Ganzi Prefecture, Sichuan Province by Zhao et al. [19]. The areas with high prevalence might be linked to special natural environmental and socio-geographical factors influenced by the high altitude. Meanwhile, the unhealthy production lifestyle of local Tibetans might have a significant similarity due to the close geographical location, thereby forming a high-risk behavior of echinococcosis [55]. Since dogs are mobile with a certain range of activities in space, their migration led to spread and prevalence echinococcosis in neighboring areas [56]. Additionally, the “low–low” gathering areas were mostly located in townships of Jiuzhaigou, Songpan, Heishui, Mao, Li, Wenchuan, Xiaojin, Baoxing, Tianquan, Kangding, Luding, Muli, Daocheng, Xiangcheng, and Yuexi counties near the southeast in Sichuan Province. These counties did not belong to the areas with a high prevalence of echinococcosis; their altitude was relatively low; the natural conditions were superior to those of high prevalence areas; production and lifestyle were relatively healthy, hence risks were relatively minimal. These findings suggest the need to strengthen comprehensive prevention and control of echinococcosis in “high–high” gathering areas. Simultaneously, favorable factors for the low prevalence of echinococcosis should be actively explored in “low–low” gathering areas to provide a baseline reference for the prevention and control of echinococcosis in “high–high” gathering areas.

This study has some limitations. Firstly, the human population were screened for hydatid lesions by using portable B-ultrasonography. Only the abdominal lesions of CE and AE could be detected, whereas lesions in the lungs, brain, and other parts outside of the abdomen could not be found. Therefore, the prevalence determined in the survey among people may be lower compared to that of the actual situation. Secondly, this study was carried out in all townships, the findings only reflect the gathering situation at the township level, so the human prevalence at the village level was still unknown. Although studies on limited spatial distribution of echinococcosis have been conducted in Sichuan Province, providing a theoretical and practical reference for control strategy making in the future, more precise spatial aggregation of the prevalence of echinococcosis in Sichuan is needed to verify and explore based on the constantly updated data and spatial analysis at village level.

## Conclusions

This study reported the echinococcosis prevalence and spatial distribution characteristics in human population at the township level in the endemic areas of Sichuan province. Human prevalence of echinococcosis was clustered in space, and the specific clustering areas were identified. Therefore, we suggest to study and formulate different prevention and control strategies in the “high–high gathering areas” and “low–low gathering areas” in the future, so as to effectively control the epidemic of echinococcosis in Sichuan Province, Thereby reducing the harm of echinococcosis to the people in epidemic areas.

## Data Availability

If data is needed, please contact the corresponding author.

## Abbreviations

AE: Alveolar echinococcosis
CE: Cystic echinococcosis
DALY: Disability adjusted of life years
LISA: Local indicators of spatial association
PE: Polycystic echinococcosis
